# PLA/PLGA-Based Drug Delivery Systems Produced with Supercritical CO_2_—A Green Future for Particle Formulation?

**DOI:** 10.3390/pharmaceutics12111118

**Published:** 2020-11-20

**Authors:** Gauri Gangapurwala, Antje Vollrath, Alicia De San Luis, Ulrich S. Schubert

**Affiliations:** 1 Laboratory of Organic and Macromolecular Chemistry (IOMC), Friedrich Schiller University Jena, Fürstengraben 1, 07743 Jena, Germany; gauri.gangapurwala@uni-jena.de (G.G.); antje.vollrath@uni-jena.de (A.V.); alicia.de.san.luis.gonzalez@uni-jena.de (A.D.S.L.); 2 Jena Center for Soft Matter (JCSM), Friedrich Schiller University Jena, Philosophenweg 7, 07743 Jena, Germany; 3 POLYMAT and Kimika Aplikatua Saila, Kimika Fakultatea, University of the Basque Country UPV/EHU, Joxe Mari Korta Zentroa, Tolosa Hiribidea 72, 20018 Donostia-San Sebastián, Spain

**Keywords:** drug delivery systems, polymeric microparticles, polymeric nanoparticles, polylactic acid (PLA), poly(lactic-*co*-glycolic) acid (PLGA), supercritical carbon dioxide (SC-CO_2_)

## Abstract

Supercritical carbon dioxide (SC-CO_2_) can serve as solvent, anti-solvent and solute, among others, in the field of drug delivery applications, e.g., for the formulation of polymeric nanocarriers in combination with different drug molecules. With its tunable properties above critical pressure and temperature, SC-CO_2_ offers control of the particle size, the particle morphology, and their drug loading. Moreover, the SC-CO_2_-based techniques overcome the limitations of conventional formulation techniques e.g., post purification steps. One of the widely used polymers for drug delivery systems with excellent mechanical (T_g_, crystallinity) and chemical properties (controlled drug release, biodegradability) is poly (lactic acid) (PLA), which is used either as a homopolymer or as a copolymer, such as poly(lactic-*co*-glycolic) acid (PLGA). Over the last 30 years, extensive research has been conducted to exploit SC-CO_2_-based processes for the formulation of PLA carriers. This review provides an overview of these research studies, including a brief description of the SC-CO_2_ processes that are widely exploited for the production of PLA and PLGA-based drug-loaded particles. Finally, recent work shows progress in the development of SC-CO_2_ techniques for particulate drug delivery systems is discussed in detail. Additionally, future perspectives and limitations of SC-CO_2_-based techniques in industrial applications are examined.

## 1. Introduction

The targeted treatment of diseases with drug-loaded carriers is becoming a substantial reality in clinical practice [1]. Diverse nano- and micrometer-sized systems based on liposomes, polymeric nanoparticles (NPs), dendrimers, and micelles are on their way to change modern medicine. The sustainability of the production of these systems, however, is still a challenge.

Conventional production strategies for the preparation of drug-loaded liposomes [2,3] or particles [4,5] usually involve organic solvents, such as tetrahydrofuran or ethyl acetate, which are often considered a health risk and contribute to environmental risks [6]. Thus, they have to be removed from the final product, together with the excess of stabilizers, by additional purification or cleaning procedures [7] which is usually followed by freeze-drying or lyophilization that leads to dry powdered product [8]. Consequently, there is an increasing demand for efficient alternative strategies that rely on sustainable chemistry and can replace (potentially) toxic organic solvents, and which additionally, offer advantages in biodegradation, waste reduction, time and energy efficiency, and in the use of renewable raw materials [9,10]. One method that is attracting significant attention in recent years is the supercritical fluid (SCF) technology, in particular the liposome and particle formulation with supercritical carbon dioxide (SC-CO_2_) [11]. SC-CO_2_ is the most economical, chemically stable, and benign compound in its supercritical state, and of high interest to replace common organic solvents.

This review describes the properties and applications of SC-CO_2_ as solvent, anti-solvent, and extraction compound for the production of drug-loaded particles, focusing on poly (lactic acid) (PLA) and its copolymer system, poly (lactide*-co*-glycolide) (PLGA). These are among the most established polymers in the pharmaceutical field [12,13]. PLA is a biocompatible aliphatic homopolymer that is approved by the food and drug association agency (FDA) for a wide range of biomedical and pharmaceutical applications, such as bone replacements scaffolds, sutures and stable particle systems [14,15]. It has two enantiomeric forms, L- and D-, along with an optically inactive meso-form. PLA can directly be synthesized via different methods such as polycondensation, ring-opening polymerization or enzymatic polymerization of the lactic acid monomer, whereby the ring-opening polymerization provides the best control over the polymerization, yielding higher molar mass PLA [16]. The latter allows tuning the stereo-chemistry of the L- and D- forms in the final polymer, which influences the molar mass and dispersity, the mechanical and thermal properties of PLA, particularly crystallinity, and the degradation behavior [16]. Additionally, it also can facilitate the tuning of the melting temperature (T_m_) and the glass transition temperature (T_g_) by varying the content of L-form of PLA [16]. The pure L-PLA (so-called PLLA) has attracted much attention from the pharmaceutical industry because of its high crystallinity. The naturally occurring degradation of PLA occurs via hydrolysis and by breaking of the ester bonds due to enzymatic degradation. This property renders it as a suitable polymeric carrier for drug molecules in nanomedicine. Furthermore, its high elasticity and tensile strength make it suitable for biomedical replacement devices and tissue engineering [16].

The use of lactic acid in combination with glycolic acid (GA) yields PLGA, which also has a contribution in this review as copolymer derivative of PLA. PLGA systems are extensively studied as sustainable drug delivery systems, thanks to their biocompatibility, biodegradability, particle size, morphology, and drug release properties in various in vivo and in vitro systems [17]. Its polymeric properties, such as the degradation rate and T_g_, can be fine-tuned by adjusting the LA:GA content ratio [7,18,19].

This review provides an overview of PLA/PLGA-based drug delivery particle systems produced with SC-CO_2_ techniques till the present date (November 2020), including a detailed description of the latest studies published since 2014 with details of polymer characteristics, encapsulated drugs, and rationales of the studies.

## 2. Carbon Dioxide as Supercritical Fluid

**Figure 1:**
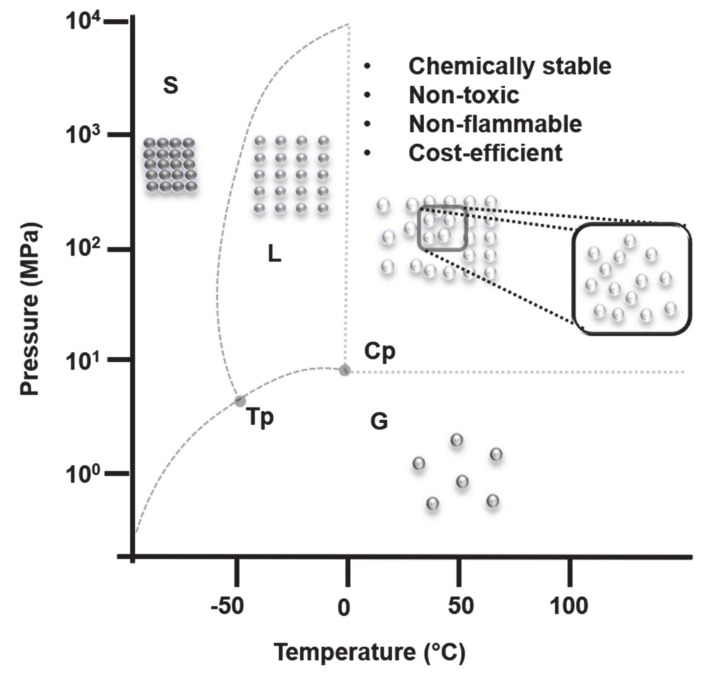
Phase diagram of carbon dioxide (CO_2_) (not to scale). S, L, and G denote the solid, liquid and gaseous phases respectively. Cp denotes the critical pressure point (31 °C, 7.3 Mpa) and Tp denotes the triple point (−56 °C, 0.5 MPa) for CO_2_.

SC-CO_2_ is presently very popular as a green alternative for several applications including extraction, production of polymers, removal of residual solvents or monomers and formulation of polymeric particles, foams or scaffolds [20]. The word “supercritical” defines the state of the solvent, which is a prerequisite for using it in the areas mentioned above. This physical state was already described in 1822 by Cagnaird de la Tour, who observed the disappearance of the boundaries between the liquid and gaseous phases when increasing the temperature far above the boiling point of water [21]. The author described the critical pressure and critical temperature concepts, including the critical point (Figure 1). SCFs impart valuable physicochemical properties such as low viscosity, high diffusivity, and high compressibility that are a combination of the liquid and gaseous states of the compound and are largely influenced by a slight change in pressure and temperature. The generation of SCFs is a reversible process; i.e., SCF goes back to liquid or gaseous phase depending on the pressure and temperature of the system. Examples of commonly known SCFs are CO_2_, acetone, chlorodifluoromethane, ethanol, hexane, propane, pentane, toluene as well as water. The supercritical conditions for acetone, ethanol, propane, toluene, and water are hard to achieve due to the high pressure and temperature required. Moreover, water in its supercritical state is highly corrosive and harsh [22]. On the contrary, CO_2_ can achieve its supercritical state at “mild” conditions, T = 31 °C and P = 73 bar. Additionally, it is non-toxic, chemically stable, non-flammable, presents low surface tension and is cost-effective.

## 3. Solubility of Polyesters in SC-CO_2_

**Figure 2:**
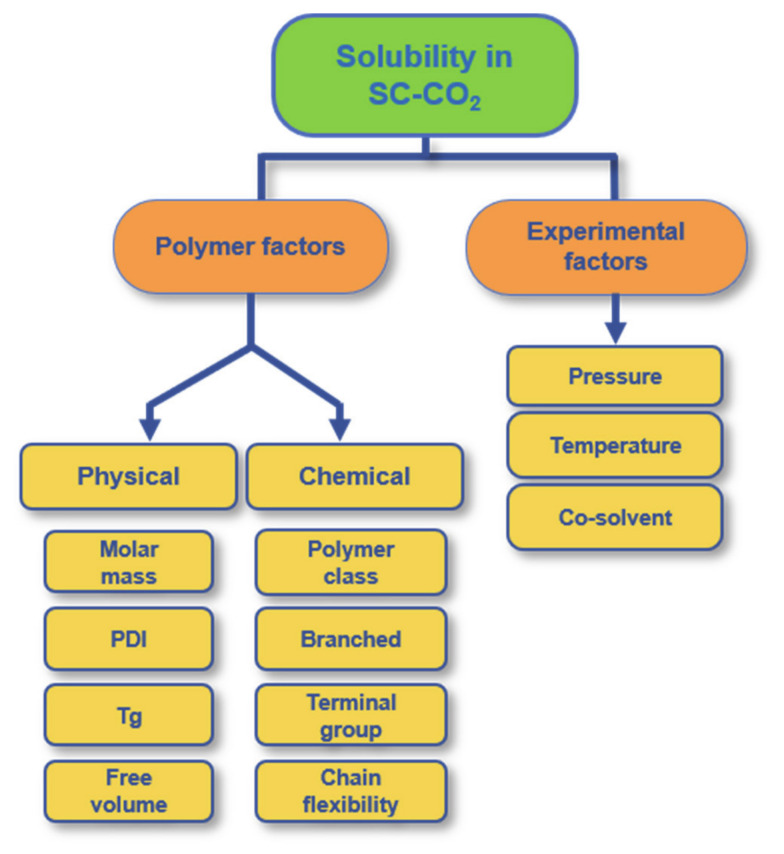
Parameters that influence the solubility of a polymer in SC-CO_2_ divided into the two main categories “polymer factors” and “experimental factors” with their illustrated subgroups.

In the light of prior research and studies, SC-CO_2_ is an established solvent for small molecules but not for organic polymers. Also, SC-CO_2_ is nonpolar and aprotic and hence exhibits certain selective solubility for ionic and polar compounds as well as for higher molar mass polymers [23]. The solubility of a polymer in SC-CO_2_ depends on its chemical structure as well as on its physicochemical and mechanical properties, e.g., molar mass, end groups (e.g., fluorine, amine), the architecture (branched or linear), T_g_ and crystallinity. However, it also depends on the experimental conditions, such as pressure, temperature, and the use or not of a co-solvent in the system. The most important parameters influencing the solubility of a polymer in SC-CO_2_ are presented in Figure 2. Moreover, the main parameters for the solubility of polyesters are briefly discussed in the following.

Compared to other polymer classes such as fluoropolymers, polyesters such as PLA generally reveal a limited solubility in SC-CO_2_, which was explained among other factors by their lower chain flexibility [23]. Nevertheless, the solubility of the polyesters can be positively influenced, e.g., by decreasing the molar mass and dispersity of the polymer. It was demonstrated that a high molar mass PLA with M_w_ = 128 kDa was not soluble in SC-CO_2_ even at high-pressure conditions, above P = 1398 bar [24]. However, it was possible to process a low molar mass PLA with M_w_ = 5.5 kDa in SC-CO_2_ at P = 250 bar and T = 65 °C without the addition of any co-solvent, e.g., for the formulation of particles [25]. Additionally, the introduction of terminal groups, such as carboxyl, hydroxyl, acetate, or fluorine groups that modify the interactions between the polymer and the CO_2_ molecule, plays an important role in the solubility properties of the polymer. For instance, J. Gregorowicz et al. confirmed that there is an influence of molar masses and terminal groups on the solubility by illustrating the difference of solubility between two different molar mass PLLA polymers with two different terminal groups. The study suggests that for low molar mass systems, the solubility is predominantly controlled by the end groups rather than by the molar mass [25].

Furthermore, the architecture of the polymer and subsequently its free volume plays a vital role in the solubility in SC-CO_2_. The free volume refers hereby to the free spaces left in the solid state of the polymeric chain at the molecular level [26]. The free volume of the polymer chain is directly proportional to the polymer chain flexibility, and leads to higher solvent diffusion and enhanced solubility in the SC-solvent. Influenced by the architecture, the free volume of a highly branched polymer is higher and, thus, it is more likely to be soluble compared to a linear polymer [27,28]. However, even if the polymer is branched, it was found that with increasing dispersity the solubility decreases, independent of the molar mass [23]. The solubility difference between linear and hyperbranched polymers, including polyesters, is not yet studied with similar molar masses. However, it is reported that the solubility of hyperbranched polymers can be positively influenced by a shorter chain length including the addition of several acetate or fluorine moieties as terminal groups [29,30].

Prior literature also emphasized that the T_g_ and the crystallinity are both related to the free volume of the polymer (T-T_g_). Therefore, lower T_g_ corresponds to higher chain flexibility of the polymer and a higher free volume which in turn, corresponds to an increased solubility [27]. Additionally, the stereochemistry represents an important factor for the solubility: semi-crystalline PLLA presenting a T_g_ of 55 to 60 °C and amorphous PDLA with a T_g_ of 50 to 60 °C have different mechanical properties and different solubility behavior. Some authors have also recognized that the T_g_ tends to decrease with increasing the pressure and the temperature, which makes the polymer more susceptible to solvation by SC-CO_2_ [16]. Moreover, the self-interactions within the polymer structure also contribute whereby, the solubility in SC-CO_2_ is higher if the self-interactions are weaker compared to the interactions between the SC-CO_2_ and the polymer [23,27]. In principle, the intermolecular and intramolecular interactions are controlling the process of mixing and solubility. Finally, the solubility of a polymer is determined by the density of SC-CO_2_, which is dependent on the process parameters and tends to elevate with pressure. With increasing pressure, the polymer solubility is likely to be enhanced.

However, if the polymer remains insoluble in SC-CO_2_, even under high-pressure conditions, polar co-solvents such as ethanol or acetone are used. The addition of these solvents contributes to decrease the difference in the free volume between the polymer and the SCF solvent and, hence, to increase the solubility of the polymer in the system [27].

Moreover, the thermodynamic equations of state (EOS) (e.g., Sanchez-Lacombe EOS) are extensively applied, in combination with the experimental protocols, to predict the solubility of polymers in SC-CO_2_ [31,32]. Predicting the conditions under which the polymer will dissolve might help to obtain a basic understanding of the solubility and SC-CO_2_/polymer interactions. Likewise, efforts to study the phase behavior of various polymers-SCF have been attempted [28,33].

At this point, there are still open questions about the solubility of polymers in SC-CO_2_. However, the SC-CO_2_ is exploited for PLA and its copolymer PLGA to obtain drug-loaded carriers. In the next sections, various processes using SC-CO_2_ to produce polymeric particles will be described in detail.

## 4. SC-CO_2_ Techniques and Important Formulation Parameters

### 4.1. Rapid Expansion of Supercritical Solution (RESS)

Early studies on the RESS process were reported already in the late 1980s. This technique is based on the rapid depressurization of supercritical solutions containing the solute, SC-CO_2_ acts as a solvent. The solute in the process can be either inorganic or organic materials, from which morphologies such as particles, thin films and powders can be attained [34,35]. The development of the process was supported by mathematical modelling and experimentally proven by J. W. Tom and P. Debendetti, who confirmed its potential to formulate polymeric monodisperse particles [31,36]. Kim et al. further illustrated the possibilities to use the RESS technique for the formulation of drug-loaded particles by the co-precipitation of biodegradable PLLA with drugs, such as naproxen. A wide range of sizes from 10 to 75 µm and especially, naproxen microparticles of 2 to 5 µm attached to >20 µm sized PLLA particles were obtained [37]. Additionally, seminal contributions made in the past twenty years suggest that the RESS technique can be exploited for the micronization of various hydrophobic pharmaceuticals to improve their dissolution behavior with the advantage of no residual solvent in the final product [38,39].

**Figure 3:**
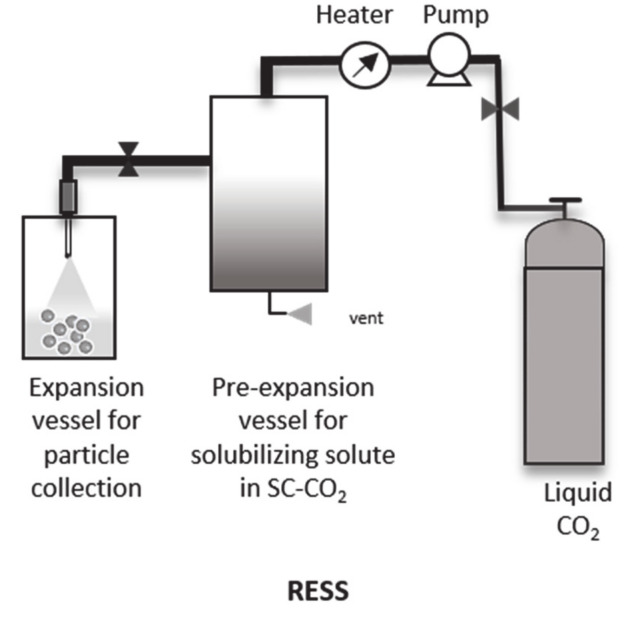
Schematic representation of the RESS process.

The RESS technique is, theoretically, a single-step solvent free (or minimal amount of organic co-solvent) particle production process, based on the high-speed expansion of the solubilized mixture of solute (polymer or polymer + drug) and the supercritical solvent (Figure 3). The process thermodynamically favors adiabatic conditions within the system [31]. However, the theoretical principle can be divided into two significant steps. At first the solute (e.g., drug or polymer) is supersaturated under supercritical conditions with SC-CO_2_. In the second step, the supersaturated solute solution is expanded rapidly through a small orifice into a vessel under depressurized conditions. The density of the solute and the solvent power of the CO_2_ rapidly decreases, resulting in particles/precipitate formation (polymer or drug). The solubility of the solute is of vital importance since supersaturation is the main factor for the initiation of particle nucleation and growth, and influences the final size and morphology of the particles. Therefore, and as mentioned previously in this manuscript, for some poorly soluble solutes a co-solvent can be used to improve the interaction between the solute and the solvent [40,41].

The final particle properties in the system can be controlled by varying the process parameters. Among many process parameters, one of the most widely used approaches in the existing literature is the study of the effect of pre-expansion variables, which include pressure and temperature [42,43]. Another important process variable that can influence the properties of the particles is the size of the nozzle, which is located in the expansion vessel. In particular, the nozzle length to nozzle diameter ratio (L/D ratio) is important [44]. Previous theoretical modelling investigations have shown that increasing the nozzle diameter (D) the particle size decreases at constant pressure [45].

A recent modification of RESS is the expansion of the supercritical solution directly in liquid solvents, so-called RESOLV [46,47,48]. In this process, the SC-CO_2_ solution containing the solute is directly expanded in a liquid to prevent aggregation of the precipitated product. The liquid in which it is expanded may or may not contain a stabilizer.

### 4.2. Supercritical Anti-Solvent (SAS), Gas Anti-Solvent (GAS) and Modifications

The application of SC-CO_2_ as an anti-solvent was introduced in the early 1990s, to obtain particles from drugs and to encapsulate active agents like such as into polymeric carriers, which limits the RESS process [49]. Examples of anti-solvent processes are SAS, GAS, and solution-enhanced dispersion by supercritical fluid (SEDS). The principle of all anti-solvent-based processes is similar, they depend on the diffusivity of SC-CO_2_ into the organic solvent in which the solute is solubilized. Therefore, these methods exploit the low solubility of the solute in SC-CO_2_, in which the solute precipitates, and the high affinity between the organic solvent and SC-CO_2_, obtaining high mass transfer rates [50]. This causes a decrease in the density of the organic solvent containing the solute which in turn, leads to a decrease in the solvation power of the solvent and causes nucleation and precipitation of the solute in the form of particles. The organic solvent is eliminated from the system along with the SC-CO_2_ [50].

**Figure 4:**
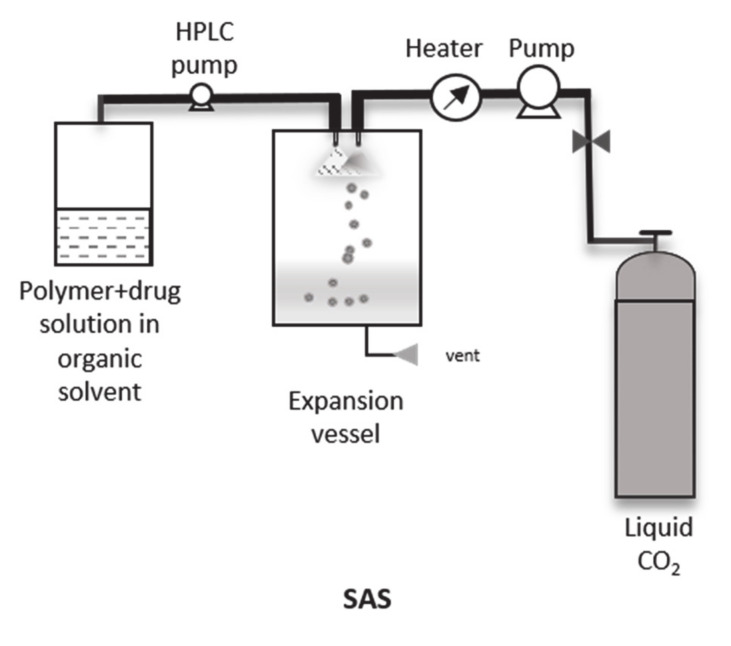
Schematic representation of SAS process.

On one hand, in GAS (batch mode) the solute-solvent mixture is poured in a high-pressure vessel and subsequently, the SC-CO_2_ is injected until the desired pressure is achieved. On the other hand, in SAS (semi-continuous mode) the organic solvent-solute mixture and the SC-CO_2_ are injected simultaneously [46,51] (Figure 4). Unlike RESS, in these cases, the mixture of phases and the precipitation of the final particles occurs in the same vessel. M. Kalani et al. and Reverchon et al. explained in depth the influence of the pressure, temperature, initial concentration of the solution, the type of organic solvent and the polymer:drug ratio on the process and final product [50,51,52]. In particular, small drug molecules can be easily processed using SAS. For example, various antibiotics (griseofulvin, ampicillin, amoxicillin, and tetracycline) and organic solvent combinations (*N*-methyl pyrrolidone, dimethyl sulfoxide, ethyl alcohol and methylene chloride) were tested [39,53]. The authors demonstrated that the choice of the organic solvent is one of the crucial factors for the success of particle formation via anti-solvent techniques. However, these techniques are not limited to the pure drug application and can be also applied for the formulation of drug-loaded polymer particles, as shown by Salmaso et al. and M. Kalani et al. [54,55]. Similar to the RESOLV modification for RESS, is the supercritical assisted injection in liquid anti-solvent (SAILA) process for SAS since the final product is obtained in suspension [56].

Finally, in the solution-enhanced dispersion by supercritical fluids (SEDS) method two co-axial nozzles are used and SC-CO_2_ is employed as both anti-solvent and dispersing agent [57]. This allows the possibility of using two types of solutions (aqueous and organic) simultaneously, facilitating the encapsulation of water-soluble drugs, such as 5-fluorouracil (5-Fu) or morphine, in a polymeric system [58,59]. The miscibility between the aqueous phase and SC-CO_2_ during the process can be increased by adding an aqueous phase-miscible modifier (e.g., ethanol) soluble in SC-CO_2_. However, one of the most important factors in the process is the ratio between the flow rates of the organic solution, the aqueous solution and the modifier which has to be fine-tuned [59].

### 4.3. Particles from Gas Saturated Solutions (PGSS)

**Figure 5:**
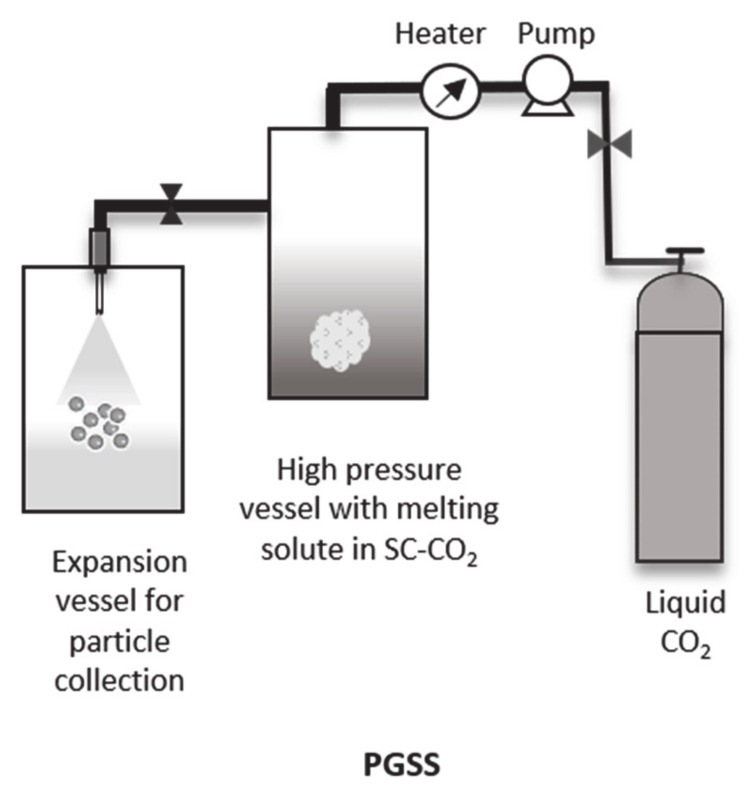
Schematic representation of PGSS process.

PGSS is a well-established technique for the production of particles or drug carriers from polymers that are capable of absorbing SC-CO_2_ but are not easily soluble as solutes. Few commonly known solutes include polyesters (e.g., PLLA and PLGA), poly(vinylpyrrolidone) and poly(ethylene glycol) (PEG) [57]. In this case, the SC-CO_2_ acts as solute and is supersaturated by the melted mixture of the polymer and the drug [29,60] (Figure 5). The supersaturated gas is expanded and the molten polymer-drug material is precipitated based on the Joule–Thompson effect [61].

### 4.4. Supercritical Fluid Extraction Emulsions with SC-CO_2_ (SFEE)

**Figure 6:**
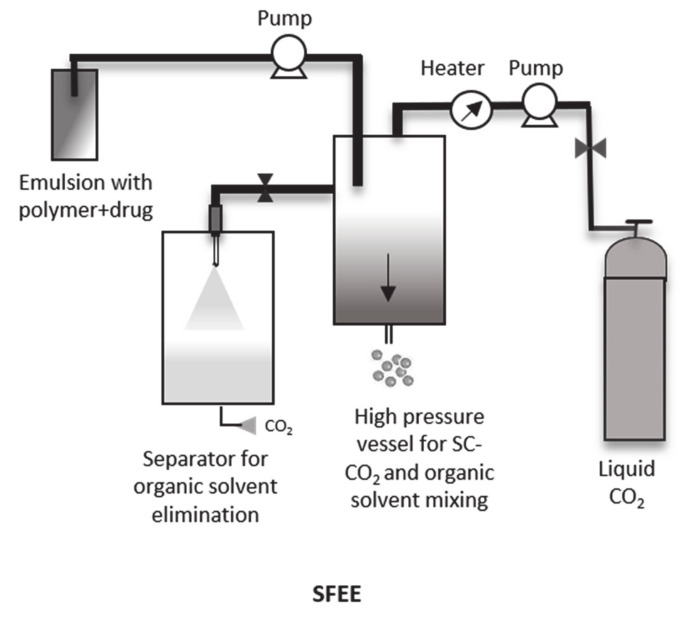
Schematic representation of SFEE process.

Finally, the SFEE method exploits the solubility of organic solvents in SC-CO_2_, whereby the diffusion and solubility depend on several parameters, including the temperature and pressure of the system [62]. In this process, a polymer-drug emulsion is formed, and the organic solvent is extracted with SC-CO_2_ (Figure 6). The advantage of this process relies on the combination of the conventional emulsion process followed by subsequent solvent extraction using SC-CO_2_. The final particle size depends on the emulsion droplet size which at the same time, depends on the technique used for generating the droplets, sonicator, or homogenizer [63,64].

### 4.5. Advantages and Limitations of the SCF Techniques

In the previous sections, the techniques in which SC-CO_2_ can be used as solvent, anti-solvent or solute were described in detail together with some representative examples. To complete this analysis, Table 1 shows a comparison of all these techniques focusing on the advantages and limitations of each of them.

## 5. Drug-Loaded PLA/PLGA Particles Using SC-CO_2_

The previous section was intended to provide a basic understanding of the principles of SC-CO_2_-based techniques. This section, on the other hand, serves as an overview of completed studies related to the production of drug delivery systems consisting of PLA/PLGA-based polymers until recently. The use of SC-CO_2_ for the formulation of polyester particles was first attempted by J. W. Tom and P. G. Debenedetti in 1991. Since then, many advances have been made in the formulation of drug-loaded polymeric systems. A summary of drug-loaded PLA and PLGA-based particles prepared via SC-CO_2_ is shown in Table 2. Furthermore, those studies that were published from 2014 are described in detail in the following pages.

### 5.1. Drug-Loaded Particles Using SAS

In 2014, A. Montes et al. exploited SAS for the encapsulation of the anti-inflammatory drugs ibuprofen and naproxen into PLLA and Eudragit (a methacrylate-based copolymer) particles using dichloromethane (CH_2_Cl_2_) and acetone as solvents, respectively [97]. For ibuprofen-loaded particles, mild operating conditions with P = 120 to 200 bar and T = 40 to 50 °C were applied. Although the conditions were similar, the Eudragit particles revealed a much smaller size, in the range of 160 ± 0.03 nm, compared to the loaded PLLA particles, with a size of 1.3 ± 0.7 µm. The maximum achieved ibuprofen loading was similar in both cases with 10% for PLLA and 8% for Eudragit. It was further observed that as the polymer to drug ratio increased (from 1:1 to 1:5), the final drug load decreased and the particle size increased. Similar results were shown for naproxen-loaded PLLA and Eudragit particles [98]. For PLLA particles, sizes between 600 nm to 1.43 µm and 13% as the highest drug loading were reported.

In a further development of the SAS process, F. Zahibi and co-workers aimed for uniform particles with smaller size distribution and an increased drug loading along with a high particle yield. The authors proposed a sonication step before the expansion of the mixture, during the mixing of SC-CO_2_ and the solvent [100]. The intermediate mixing supported the mutual diffusion and combination of the solvent and the solute achieving a higher supersaturation, which resulted in smaller and more uniform particles. The authors investigated the influence of several variables, such as the flow rate of the organic mixture, the polymer:drug ratio and the ultrasonication power (W), on the yield, the drug loading, and the size of the final product. They observed that an increase in the solution flow rate resulted in a decrease in the mean size of the particles but in an increase of the drug loading. A similar effect was observed when the ultrasonic power increased from 120 to 240 W. This increase in the ultrasonic power enabled an efficient mixing that led to a higher mass transfer. However, if the ultrasonic power was further increased to 300 W, the opposite effect occurred and the average size of the particles increased and the drug loading decreased. This was explained as a result of the heat generation due to the high ultrasonic power. Further influencing parameters such as the static agitation time or the influence of the molar ratio of CO_2_ to organic solvent were explored. The increase of the static agitation time to six seconds considerably affected the loading compared to non-static agitation. In addition, a higher molar ratio of CO_2_ to solvent led to smaller and more uniform particles due to higher supersaturation.

In 2015 and 2018, anti-solvent techniques similar to SAS were applied to produce insulin-loaded PLLA porous microspheres, as reported by A.-Z. Chen et al. and Lin et al. [103,110]. The former group used a modification of a process described in 2007. This procedure was based on the spraying through a coaxial nozzle of the polymer-drug solution, previously pressurized with SC-CO_2_ in a second high-pressure vessel, causing the formation of droplets due to supersaturation [112]. In the work reported here, the authors first formed an emulsion with PLLA dissolved in CH_2_Cl_2_ and insulin dissolved in the water phase. The authors obtained particles with an aerodynamic diameter of 4.31 µm. In the latter study, an insulin loading of around 5% and encapsulation efficiency (EE) of 97% was reported by subjecting the INS-PLLA emulsion to the process described above. The conditions applied for the process were P = 80 bar and T = 35 °C using a flow rate of 4 mL/min. The resulting particles revealed a rough surface and a mean diameter of 16 ± 3 µm if no drug was present, whereas they were found to be slightly larger, with a size of 19 ± 3 µm, when insulin was encapsulated. Subsequent in vitro and in vivo studies proved the biocompatibility and the desired hypoglycemic effects for pulmonary delivery of the prepared insulin-PLLA particles with a sustained drug release.

In another study, anti-retroviral drugs presenting low permeability, high dissolution rate and low bioavailability such as zidovudine (3′-azido-2′3′-dideoxythymidine) were considered for their encapsulation into PLLA using SAS, to obtain solid dispersions that improve intestinal absorption [102]. Briefly described, three different zidovudine:PLLA ratios (2:1, 1:1 and 1:2) were formulated in ethanol using three different pressures (P = 135, 100 and 85 bar) while keeping the temperature constant at 45 °C for all runs. At 85 bar the loading capacity (LC) of the particles was calculated to 13%, 23% and 7%, respectively. Although the EE was higher for the drug:polymer ratio of 1:2, ex vivo experiments that evaluated the intestinal permeability proved higher zidovudine permeation for the 1:1 ratio. This result indicates that the use of SAS method allows the modification of suitable polymeric carrier systems in terms of absorption and dissolution properties of the drug [102].

More recently, P. S. C. Sacchetin et al. attempted the production of PCL/PLA particles for the controlled release of the hydrophobic steroidal drug 17α-methyltestosterone with SAS [104]. The influence of the PCL content in the PCL/PLA particles and of different ratios of steroidal drug to PCL/PLA ratio were studied at P = 800 bar and T = 40 °C. With increasing the amount of PCL from 1:9 to 1:1 (ratio related to PLA) the size of particles increased up to 53 µm. Likewise, the EE increased from 20 to 51%. On the other hand, a higher amount of 17α-methyltestosterone in the system led to amorphous powder formation with an increase in the mean diameter from 23 to 54 µm. The release of the steroid from the particles was proven at different pH values simulating gastrointestinal conditions.

The successful production of a controlled delivery system with tamoxifen (TAM) as payload using SAS at P = 130 bar and T = 38 °C was further reported by D. Alias and co-workers [107]. To confirm the chemical and physical stability of the drug before and after the process, the authors compared the characteristics of unprocessed TAM, encapsulated TAM, and micronized TAM. The Fourier transform-infrared (FTIR) data revealed that the SAS process did not affect the chemical composition of TAM and that no free TAM was observed in the encapsulated sample. Regarding the morphology, TAM loaded particles were spherical with a smooth surface and amorphous compared to the unprocessed TAM, indicating an improvement of the NP dissolution in water. Finally, the product was free of residual solvent confirming the potential of SC-CO_2_ in the production of drug delivery systems with no post formulation purification steps needed. Similar studies with gefitinib as anti-cancer drug have been attempted using SAS to obtain gefitinib loaded dry powdered inhalers [109]. Lin et al. optimized thereby various system parameters, such as the solvent:co-solvent ratio of CH_2_Cl_2_ and EtOH (ranging from 0 to 0.5), the concentration of PLLA, the flow rate of the polymer-drug solution (F = 0.5 to 1.6 mL/min), as well as the pressure (P = 90 to 120 bar) and the temperature (T = 33 to 48 °C). The flow rate revealed the highest impact on the final gefitinib loading, and at optimized conditions (CH_2_Cl_2_/EtOH ratio = 0.2, c= 5 mg/mL, F = 1.2 min/mL, T = 48 °C and P = 90 bar) spherical particles with an average diameter of 2.5 µm, a LC of 16% and an EE of 94% were obtained. It was further shown that the crystallinity decreased compared to unprocessed gefitinib and that no solvent traces were left in the final product. Gefitinib loaded PLLA MPs revealed that a sustained release over the time was more effective at lower concentrations compared to raw gefitinib.

A recent study used a mixture of dimethyl sulfoxide (DMSO) and CH_2_Cl_2_ (ratio 1:1 *v*/*v* %) within the SAS process for the production of PLLA-coated 5-Fu [111]. The temperature-pressure conditions for various runs were 35 and 50 °C and 120, 150 and 180 bar, keeping the other process parameters such as the solution concentration and flow rates, unchanged. The obtained size for the coated particles varied from 0.6 to 1.2 µm (at 35 and 50 °C respectively) and the drug load ranged from 32 to 42%, whereby the best results were observed at 50 °C and 180 bar. The dissolution profile clearly showed a slower release rate from the PLLA-coated 5-Fu particles compared to the micronized particles.

### 5.2. Drug-Loaded Particles Using Modified SAS

**Figure 7:**
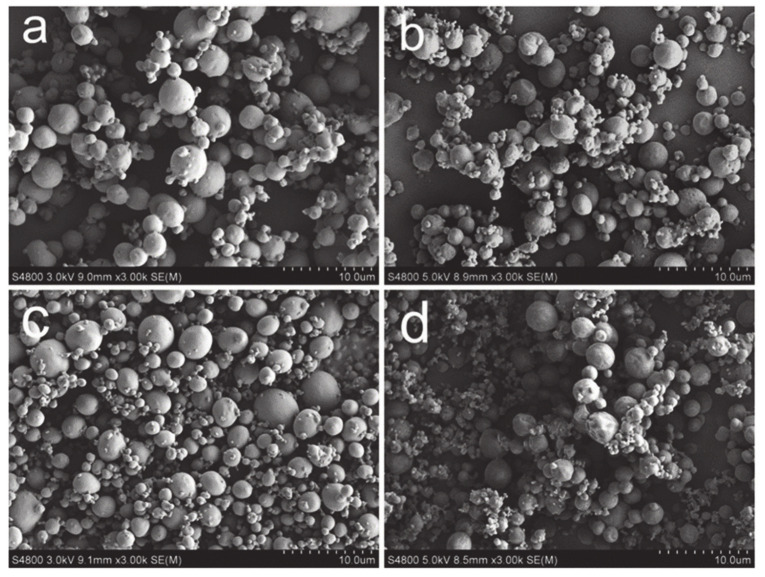
SEM images of microparticles prepared using the SEDS process. (**a**) FA-PEG-PLLA, (**b**) paclitaxel-loaded FA-PEG-PLLA (90% FA), (**c**) PEG-PLLA and (**d**) paclitaxel-loaded PEG-PLLA (scale 10 µm). Reprinted with permission from [101], Springer Nature, 2015.

Although the conventional SAS technique proved to be promising over the years for drug-loaded PLA-based delivery systems, many authors also used SEDS and the modified SAS technique SAILA for this goal. The SEDS technique that uses two co-axial nozzles and enables the simultaneous introduction of two different solutions was used to formulate PLLA-based particles with and without the presence of drugs, e.g., morphine, 5-Fu and paclitaxel [59,91,101]. Paclitaxel was encapsulated into PEG-PLLA and folate (FA) conjugated FA-PEG-PLLA polymer with varying the content of FA (0.3, 0.60 or 0.9 to 1.0) using CH_2_Cl_2_ as organic solvent [101]. Scanning electron microscopy (SEM) images of paclitaxel-loaded and -unloaded particles are displayed in Figure 7. The final particle sizes ranged between 2.3 and 3.5 µm. The highest EE was 23% and was obtained with the lowest drug:FA-PEG-PLLA ratio of 0.3:1. However, it was reported that the ratio of 0.6:1 with encapsulated paclitaxel proved to be more effective in vivo due to the targeting effect.

**Figure 8:**
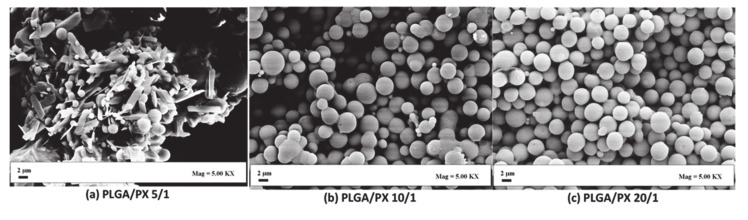
Piroxicam-loaded PLGA particles produced by SAILA with varying polymer/drug ratio of (**a**) 5:1 (**b**) 10:1 and (**c**) 20:1 (scale 2 µm). Reprinted with permission from [108], Elsevier, 2017.

On the other hand, SAILA was applied by Campardelli et al. to demonstrate the successful production of an aqueous suspension of PLGA MPs loaded with the anti-inflammatory lipophilic drug piroxicam [105]. In preliminary experiments, the authors used PLGA and PLA as carriers and studied different process parameters, such as temperature (T = 60 to 80 °C), gas to liquid ratio (G/L ratio = 1 to 2.6) as well as various polymer concentrations at constant pressure (P = 80 bar). Spherical, smooth particles of both polymers in the sub-micron region (d = 0.4 ± 0.1 μm) and micron region (2.1 ± 0.8 μm) were produced, whereby the final particle size decreased from the micro-region just by increasing the G/L ratio to 2.6. Piroxicam-loaded PLGA particles with a size of 1.3 µm ± 0.3 and an EE of 60% could be produced by using a PLGA concentration of 2.5 mg/mL and a polymer: drug ratio of 1:20 (settings: P = 80 bar, T= 80 °C, G/L ratio = 1.6). R. Campardelli et al. also proposed the same technique to co-precipitate the anti-inflammatory drugs indomethacin and diclofenac, additionally to piroxicam with PLGA at 80 bar, 60 °C and using a gas to liquid ratio of 1.4 [108]. Particles in the range of 2 to 3 µm with an EE between 64% and 86% were obtained for all active payloads. The piroxicam-loaded PLGA system was further studied with varying the polymer:drug ratio and the concentration of the polymer solution. With increasing the polymer:drug ratio (5:1, 10:1 and 20:1) the size and dispersity of the particles decreased, as represented in Figure 8. The particle size could be further decreased by reducing the initial polymer concentration from 10 mg/mL to 2.5 mg/mL. However, it was noticed that the larger particles revealed slower release rates.

### 5.3. Drug-Loaded Particles Using Supercritical Fluid Emulsion Extraction (SFEE)

Another SC-CO_2_ assisted approach adopted at present and often used to obtain drug-loaded particles, with no or low residual solvent content, is the supercritical fluid emulsion extraction (SFEE) technique that was already mentioned earlier in Section 4.4. The SFEE process is based on the formation of an emulsion and posterior subjection to SC-CO_2_ in atomized form. The event increases the contact opportunities between SC-CO_2_ and the oil phase, causing the precipitation of the polymer-drug mixture in particle form due to a mass transfer mechanism [63]. Thus, critical aspects concerning the solvent removal after formulation in conventional emulsion method can be avoided [61].

**Figure 9:**
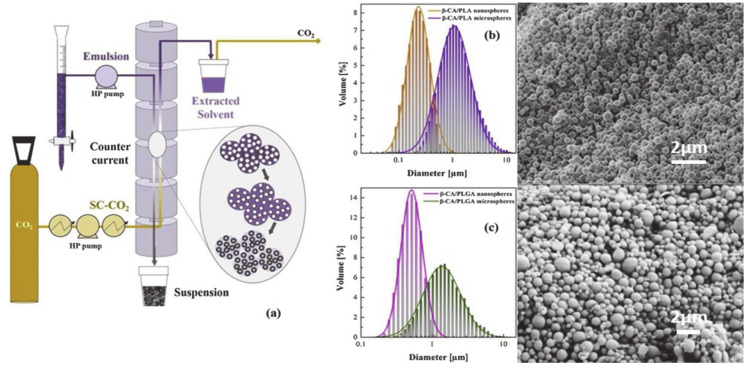
**Left side.** Schematic representation of the SFEE process (**a**). Size distributions of PLA (**b**) and PLGA (**c**) particles showing the possibility of varying particle sizes by modification of the initial droplet size in the emulsion. **Right side**: SEM images of PLLA loaded particles obtained from the emulsion after application of the SFEE method. Reprinted with permission from [113], Elsevier, 2019.

The most recent study using SFEE was published in 2019 by C. Gimenez-Rota et al. [113]. In this report, β-carotene is encapsulated together with the anti-oxidants α-tocopherol and rosmarinic acid into PLLA and PLGA particles [113]. Experiments with several different double emulsions and various external phases were shown in this study. The SFEE process was carried out at 80 bar and 37 °C throughout all the experiments with a constant liquid-gas ratio of 0.1. The final sizes for the loaded PLLA particles ranged from 0.5 to 1.4 µm, whereby the influence of the droplet size of the emulsion on the final particle size was successfully demonstrated (see Figure 9). If the droplet size in the PLLA emulsion was about 2.1 µm, a final particle size of 1.4 µm was obtained. For a droplet size of 1.2 µm in the emulsion, the final PLLA particles revealed a size of 0.3 µm. The EE was calculated to be 72% and 62%, respectively. For PLGA, the same droplet sizes (2.1 and 1.2 µm) were tested and particles of 2 and 0.3 µm with an EE of 52% and 62%, respectively, were reported. The authors also mention that the use of a water-miscible solvent such as acetone failed to provide a stable emulsion and concluded that the SFEE process depends on the solvent and the carrier selected in the system. An overview of further research studies which used the SFEE technique for the removal of the organic solvents is presented in Table 3.

## 6. Industrial Application of Using SC-CO_2_ Processes for the Production of Drug-Loaded Particles

The supercritical fluid-based technologies are categorized as the bottoms-up approaches for the formulation of drug delivery systems in environmentally friendly conditions. Although the process seems simple, previous work has shown that there cannot be universally applicable conditions and that they must be specifically optimized for each polymer/drugs system. To date, SC-CO_2_ is the best choice as SCF for the pharmaceutical industry, but the SC-CO_2_-based techniques raise questions that are not yet answered for scale-up processes, e.g., the GMP cost estimation, calculation for estimation of parts of GMP equipment, together with the above-mentioned lack of standard processing conditions [121].

Theoretical modelling predictions try to find the answer to understand the phase equilibria of a system with multiple components. These studies could also answer the questions of the compatibility/solubility of the solutes in the SCF, make hydrodynamic modelling of the fluid flow through the capillary and microparticle nucleation and help in the development to have better control over the final product properties. However, this is still in progress and additionally, these theoretical answers also need experimental data to correlate the predictions with the experimental results and in the end, offer a suitable process for practical use [122].

So far, only a few processes such as SFEE were implemented and patented for the production of fine particles, and certainly more intensive and rigorous research is required [123,124]. A review from P. Sheth et al. has focused on the application of the SCF technologies for NP systems in the pharmaceutical industry. The authors nicely presented various industrial-scale leaders for the production of NPs which also includes SFC-based industries [57]. Nevertheless, it is obvious that although the advantages of the SC-CO_2_-based processes are numerous, many gaps in knowledge still need to be bridged. Thus, further investigations and evaluations of the process parameters are urgently needed to realize a broad and successful application of this technology in the near future.

## 7. Conclusions

The techniques described above demonstrate the enormous potential of SC-CO_2_ for the production of PLA and PLGA-based drug delivery systems, including solid dispersions, formulations for oral delivery as well as inhalations. SC-CO_2_ offers the advantages of being non-toxic and inexpensive and is the right step in the direction of green chemistry for the production of drug carrier systems. PLA and PLGA particles in the micro-and nanometer range can be produced by techniques that involve SC-CO_2_ as solvent (RESS, RESOLV), anti-solvent (SAS, SEDS, SAILA) or extractant (SFEE), depending on the SC-CO_2_ compatibility with the system materials and the desired product [61,79]. However, after the study of the various scientific articles, it is evident that the anti-solvent techniques are a popular choice for particle formulations. The low solubility (and low polarity) of PLA and its copolymer in SC-CO_2_ can be blamed for this challenge in techniques based on SC-CO_2_ acting as a solvent, such as RESS. Hence, the inclination toward anti-solvent techniques such as SAS, SAILA, and along with PGSS are explored and investigated owing to the advantages of SC-CO_2_.

The design and formulation of drug-loaded biodegradable particles with improved control over the system are still in progress. Exploiting the solvent/anti-solvent properties of SC-CO_2_ for the particle formation is thereby challenging. The major obstacles for wide-scale applications of SC-CO_2_-based techniques are the limited choice of soluble biopolymers, the tendency of some polymers to plasticize during processing, the compatibility of organic solvents and the technical apparatus. At the same time, the principles of the particle formation and size distributions are dependent on the solute-solvent properties and the thermodynamics of the system. There are numerous unknown variables involved in the system, which indicates the necessity for solutions for the transfer of technology or upscaling. The application of theoretical modelling tools to understand phase equilibria, which are not covered in this overview, is expected to fill the gaps in the present research [125,126].

With thorough experimental investigations and correlating the findings with appropriate theoretical modelling predictions, the SC-CO_2_-based processes can be a reality in the coming years for the production of polymer-based drug delivery systems if enough energy and focus is set on this technology.

## Figures and Tables

**Table 1:** Overview of advantages and limitations of the SC-CO2-based techniques.

SCF Technique	Advantages	Limitations
RESS, RESOLV	Single-step particle production. No/low amount of organic solvent required. Final product properties can be controlled by controlling the process parameters. The final product is free of residual solvent.	Solute should be solid or amorphous. The solubility of solute is selective for low molecular weight polymer and small molecules.
SAS, GAS, SEDS	Milder process parameters (temperature and pressure required) compared to RESS. Overcomes the limitation of solute solubility in SC-CO_2_. Encapsulation of labile active substances is possible.	Use of organic solvent. Some biopolymers tend to plasticize in presence of SC-CO_2._
PGSS	Organic solvent free process. Homogeneous product obtained. Encapsulation of labile active substances is possible.	Particle aggregation can occur during the product formation. Nozzle blockage can occur.
SFEE	Wider range of biopolymers can be processed including PCL and PMMA. Encapsulation of hydrophobic drugs, proteins and essential oils is possible. Monodisperse particle production.	Multiple steps required. Organic solvent is used.

**Table 2:** Overview of (drug-loaded) PLA/PLGA particles obtained using SC-CO_2._

SCF Technique	Polymer; Molar Mass (kDa)	Cargo/ Drug	Overview	Ref/Year
RESS, SAS	PLLA; 5.5 PDLLA; 5.3	-	Production of MPs of PLLA, PDLLA and PGA via successful processing of polyhydroxy polymers.	[36]/ 1991
RESS	PLLA; 10	Pyrene	Production of composite particles using RESS: variation of production parameters and use of pyrene as a model compound for coprecipitation with PLLA.	[65]/ 1994
RESS	PLLA; 2	Naproxen	Microencapsulation of Naproxen in PLLA. Application of specific equation of the state theory for a prediction of the parameters: EOS modeling of the process parameters was successfully correlated with the experimental results.	[37]/ 1996
ASES	PLLA; 102	Hyoscine butyl bromide, indomethacin, piroxicam and thymopentin	The Aerosol solvent extraction system (ASES) technique was used to produce drug/polymer particles using various model drugs. Drug-loaded PLLA MPs with sizes below 50 µm were obtained.	[66]/ 1996
PCA	PLLA; 100	Gentamycin, rifamycin and naltrexone	The PCA method with ion pairing as a preliminary stage was used to encapsulate drugs into PLLA. Their release profile was revealed indicating matrix-controlled diffusion for particles produced by PCA.	[49]/ 1997
SEDS	PDLLA; n/d PLLA; n/d PLGA; n/d	-	Successful preparation of PDLLA, PLLA, PLGA and PCL MPs using SEDS technique.	[67]/ 1999
GAS	PLLA; 102	Insulin	Preparation of insulin-loaded PEG-PLLA NPs with varying content and molar mass of PEG. The low molar mass PEG improved the morphology of the co-precipitation product and drug release kinetics.	[68]/ 2001
SAS	PLLA; 29	Diuron	Production of diuron loaded PLLA MPs ranging from 1–5 µm by single-step SAS process. A different concentration of diuron and PLLA in the system affected the final morphology of the product.	[69]/ 2001
ASES	PLLA; 50	4-Hydroxy-benzoic acid	Drug-loaded PLLA MPs with sizes between 2 to 3 µm and loading capacities of 9.2% and 15.6% were achieved (for *p*-hydroxybenzoic acid and lysozyme).	[70]/ 2002
PCA	PLLA; 100	Budesonide	Preparation of budesonide loaded PLLA MPs was successful. The obtained product particles showed ~80% encapsulation efficiency (EE) and were studied in vitro prior to in vitro drug release analysis.	[71]/ 2002
GAS	PLLA; 102	Insulin	Production and in vitro investigation of insulin-loaded PEG-PLLA particles with sizes <1 µm. The PEG content was varied observing that the loading capacity of insulin was inversely proportional to the molar mass of PEG.	[72]/ 2004
GAS	PLLA; 102	Nissin	Formulation of nissin loaded PLLA particles with antibacterial activity via GAS technique proving its potential for protein encapsulation.	[54]/ 2004
Pressure quenching	PLGA (50/50); 96 (65/35); 10 (75/25); 97 PLLA; 160	Deslorelin	Drug encapsulation into PLGA polymer particles of different compositions. PLGA (50/50) was found to work the best; The sizes of the drug-loaded particles ranged from 2.2 to 13.8 µm. PLLA was not used further. The technique proved to maintain the integrity of the active substance, low residual solvent and sustained release of the final product.	[73]/ 2004
SC-CO_2_ assisted plasticization and spraying	PDLLA; 8	Ribonuclease-A, lysozyme, insulin, and calcitonin	Successful encapsulation of proteins in PDLLA MPs. PDLLA was first plasticized using SC-CO_2,_ then the drug was mixed with it and the molten mixture was sprayed to obtain dry particles. The particles retained the enzymatic activity of insulin and calcitonin which was confirmed by biological assays.	[74]/ 2005
SSI	PDLLA; 9	Indomethacin	Indomethacin was impregnated on PDLLA NP carriers maintaining the size between 190 to 290 nm and spherical morphology of the particles with up to 6.6 wt% of the drug	[75]/ 2005
SAS	PLLA; 2 PLLA; 50 PLGA (50:50); 48	Bupivacaine hydrochloride	Encapsulation of bupivacaine hydrochloride in PLLA (M_W_ = 2 kDa) MPs. Varying the ratios of the PLGA/PLLA polymer content, two different levels of bupivacaine HCl (5 and 10%) were used and particles of 4 to 10 µm in geometric mean diameter were obtained. A controlled drug release was observed for up to 7 days.	[76]/ 2006
SEDS	PLLA; 100 PLGA; 100	Paclitaxel	PLLA, PLGA_(50/50)_ and mixtures of both (PLLA/PLGA_(50/50)_) were co-precipitated with paclitaxel to obtain MPs. The size of the MPs was influenced by the feed ratio. Final drug loading was between 14.1 and 16.3%. The release rate depended on the ratio of PLLA/PLGA in the final product.	[77]/ 2008
SAS	PLLA; 85–160	Rifampicin	Formulation of rifampicin loaded PLLA NPs with varying PLLA:drug ratios. The average particle size was <5 µm. A lower polymer content resulted in more irregular shaped particles, but a higher polymer content decreased the drug loading. 7 to 80% of polymer content particles were found to show a more controlled release of the drug without an initial burst.	[78]/ 2008
SEDS	PLLA; 100 PLGA; 100	Indomethacin	Indomethacin loaded PLLA/PLGA_(50/50)_ MPs with 2.35 µm size were produced. Release studies revealed diffusion-controlled release in early stage and bioerosion in later. In vitro cell studies revealed the activity of IDMC-PLLA/PLGA particles on the non-small cell lung cancer cell line.	[79]/ 2008
SEDS	PLLA;100	SiO_2_ NP 5-Fluorouracil	5-Fluorouracil was adsorbed on SiO_2_ NPs which were then co-precipitated with PLLA. Spherical smooth particles with a size of ~530 nm were obtained. The EE was claimed to be 80.53%. The technique proved to be efficient for the preparation of drug-loaded MPs with controlled release of the drug.	[80]/ 2009
SAS	PLLA; 100	5-Azacytidine	The drug was encapsulated in PLLA MPs final sizes were ~2 µm. Optimal conditions of 40 °C and 11 MPa, and a CO_2_ flow rate of 30 mL/min were used to obtain 5-Azacytidine loaded MPs with 95% drug entrapment. The drug release profiles in acid and basic medium were reported as well as the stability of the entrapped drug.	[81]/ 2009
RESOLV	PLLA; 11	Retinyl palmitate	Retinyl palmitate was encapsulated in PLLA yielding particle sizes in the range of 40 to 110 nm and an average loading of 0.9 to 6.2%. The entrapment efficiency increased with increasing the pre-expansion temperature and the concentration of drug, but the size of the NPs was affected by the degree of saturation.	[47]/ 2009
SEDS	PLLA; 50 PLLA-PEG; 70-5	siRNA	siRNA loaded L-PLA and PEG-PLA particles with a size range between 100 to 300 nm and 600 to 700 nm respectively were obtained. Furthermore, the drug release was found to be dependent on the polymer used. The in vivo sustained release was successfully studied without affecting the biological activity of the siRNA.	[82]/ 2010
SAA	PLLA; 85–140	-	Spherical MPs of PLLA were produced in the size range of 1 to 1.5 µm. BSA was also processed and spherical particles with retained biological activity were obtained. The process was hence claimed to be suitable for thermolabile compounds.	[83]/ 2011
RESS + SEDS	PLA-PEG-PLA; 29	-	The study revealed the advantages of combined SEDS and RESS technique for processing partially soluble polymers obtaining particles with sizes of ~2 µm. The process parameters controlled the morphology and size of the final product during the process.	[84]/ 2011
Reverse emulsion SEDS	PLLA-PEG; 25:5; 50:5; 100:5	5-Fluorouracil	Drug/copolymer emulsion with varying ratios of PLLA:PEG (organic phase) were formed with the subsequent SC-CO_2_ spraying. The drug-loaded particles revealed a size ≤ 1 µm and were efficient to inhibit the growth of tumor in animal studies by 51.92%, which is higher than the free 5-Fluorouracil. This technique proved to be efficacious for the encapsulation of hydrophilic compounds.	[58]/ 2012
SAA	PEG-PLA; 7.7–9.7; 8.1–23.1	-	Spherical MPs of PLLA were produced in the size range of 1 to 1.5 µm. BSA was also processed and spherical particles with retained biological activity were obtained. The process was therefore claimed suitable for thermolabile compounds.	[85]/ 2012
SpEDS	PLLA; 77	Fe_3_O_4_ NPs Methotrexate	Production of methotrexate loaded PLLA MPs via drug and polymer co-precipitation on Fe_3_O_4_ NPs was presented. Particle size decreased with increase in Fe_3_O_4_ content. The EE was 60.8% and a sustained released was observed. The SpEDS process was successful in the production of magnetic particles.	[86]/ 2012
SEDS	PLLA; 10 PLLA-PEG; 50, 100, 200	Lysozyme	Lysozyme loaded PLLA/PLLA-PEG MPs with varying sizes were produced. The particles size and drug loading were affected by the PEG content and by the M_W_ of the PLLA. However, the structure of the initial polymer did not change during the SEDS processing confirming that protein or labile compounds can be processed using this technique.	[87]/ 2012
SAS	PLLA; n/d	Paracetamol	The process parameters for the encapsulation of paracetamol into PLLA MPs were investigated. Final particle sizes varied with increasing pressure or temperature. A sustained drug release was observed over 4 weeks proving that SAS can be promising for the production of drug-loaded vehicles.	[88]/ 2012
SEDS	PLLA; 100	Morphine	Morphine loaded PLLA (MF-PLLA) spherical MPs with a mean diameter of 2.45 μm were prepared using the optimum conditions of 17.5/2.5 aqueous solution, ethanol and DCM ratio. The calculated drug loading was 4.73 ± 0.34%. The release profile revealed a burst release in first 4 h and slow-release until 168 h.	[89]/ 2012
ASES	PLLA; 50 and 100; mPEG-PLLA various ratios	Leuprolide acetate	Leuprolide acetate loaded MPs using PLLA and mPEG-PLLA were studied regarding the polymer molar mass, the block length and the drug:polymer ratio. The increase of the PEG length in the copolymer increased the particles size from 2.86 to 5.63 µm. The study showed the influence of the varying factors on the final product morphology and size.	[90]/ 2012
SEDS	PLLA; 100 PLLA-PEG-PLLA; n/d	Morphine	Morphine was encapsulated in PLLA-PEG-PLLA particles with particle size range of 2.04–5.73 µm. The influence of the process parameters and the content of PEG on the final product was studied. The highest EE of ~87% was obtained at drug:polymer ratio of 1:10 with 3% PEG and at 120 bar and morphine concentration of 8 mg/mL. The release rate was faster by increasing PEG content in the final product.	[59]/ 2013
RESOLV	PLGA (85/25); n/d	Fenofibrate	PLGA was co-precipitated with fenofibrate obtaining particles with sizes of ~3 µm. The most suitable stabilizer was SDS driving to a particles size of ~0.89 µm. Furthermore, coprecipitation of PLGA and fenofibrate was attempted with SDS as a stabilizer, where the structures obtained were comparatively non agglomerated and in the size range below 1 µm. Increasing the number of depressurization cycles resulted in the agglomeration of the final product.	[91]/ 2013
SAS	PLLA; 100	10-Hydroxycamptothecin	Encapsulation of 10-hydroxycamptothecin into PLLA NPs with subsequent in vitro release studies is shown. The loading capacity of the particles was influenced by the solvent combination used. The loaded particles produced with suitable conditions were spherical in shape with 794 nm (mass median diameter) size with a loading capacity of 13.3%. The in vitro release investigation proved that the increase in the concentration of PLLA influences the controlled release and loading capacity of the drug.	[92]/ 2013
PCA	PLLA; 50	Lysozyme	Lysozyme loaded PLLA MPs were produced with irregular shape, in a size range of 16.9 ro 18.8 µm and with a porosity of 78.2–86.3%. Further characterization also revealed no changes in the structure or activity of the active compound during the process, proving the suitable for processing and encapsulation of labile materials.	[93]/ 2013
RESS + SAS	PLLA; n/d	-	RESS and SAS techniques were applied to PLLA for the production of MPs and revealed that the technique had a major influence on the morphology and size of the particles. Spherical particles were obtained with SAS process, which was then chosen for further investigation. The size of the particles was largely dependent on the initial concentration, flow rate, process temperature and pressure in the SAS process.	[94]/ 2013
SC-CO_2_ infusion pressure quench technique	PLLA; n/d PLGA; n/d	Bevacizumab	Bevacizumab was coated on PLLA NPs that were further encapsulated into porous PLGA microparticles by exposing the mixture to SC-CO_2_. The Bevacizumab loaded PLLA particles (265 nm) were infused in porous PLGA of 1.67 µm. The sustained release proved to be effective for 4 months with retained signaling protein activity (Vascular endothelial growth factor).	[95]/ 2013
SSI	PLLA; 100	5-Fluorouracil	PLLA MPs were prepared and impregnated with 5-fluorouracil. The resulting size was 0.68 µm. The impregnation efficiency was influenced by the pressure, temperature, and co-solvent concentration. The highest impregnation efficiency observed was 12 µg/mg at 60 °C and 100 bar without cosolvent and 60 µg/mg with 4 mol % of cosolvent.	[96]/ 2013
SAS	PLLA; n/d	Ibuprofen	Co-precipitation of ibuprofen and PLLA resulting in MPs with sizes between 0.9 to 1.8 µm and up to 10.1% drug loading. It was observed that increasing the pressure and decreasing the temperature decreased the particles size. The maximum amount of drug remained on the surface of the particles, which was confirmed by release rates and FTIR, XRD and DSC analysis.	[97]/ 2014
SAS	PLLA; n/v	Naproxen	Co-precipitation of naproxen and PLLA yielded MPs with a size of 1.2 µm and 13.2% drug loading. A slow controlled release was observed compared to free drug. The pressure had an inverse effect on the final particle size. The drug:polymer ratios did not influence the release rate of the profiles and the pH value of the external medium.	[98]/ 2014
SAS-EM	PLGA; 50	Curcumin	Production of curcumin loaded PLGA particles with modified SAS whereby the polymer-drug solution was ultrasonicated before spraying to enhance the mixing which resulted in a higher drug loading and improved yield.	[99]/ 2014
SAS-EM	PLGA; 50	Curcumin	Optimization of the process parameters to obtain curcumin coated PLGA (75:25) NPs. The influence of the solvent type, the initial concentration of solute, the CO_2_ flow rates, the curcumin:PLGA ratio and the ultra-sonication power was investigated. Loading, size distribution and yield of the final curcumin coated PLGA NPs improved with increasing the ultrasonic power and high solvent flow rates.	[100]/ 2014
SEDS	PEG-PLLA; 27	Paclitaxel	Encapsulation of paclitaxel into a folic acid (FA)-PEG-PLLA copolymer for tumor targeting yielded MPs with sizes between 2.4 and 2.9 µm and a drug loading of 7 to 9%. The FA content influenced the EE obtaining the highest (23%) for a FA:polymer ratio of 0.6:1. The PTX-FA-PEG-PLLA particles showed higher uptake in tumor cell lines while the distribution of the drug was found to be specifically higher in tumor tissues. In vivo studies revealed that FA conjugated particles were more effective compared to the unconjugated particles.	[101]/ 2015
SAS	PLLA; n/d	Zidovudine	Zidovudine-loaded PLLA particles (dry dispersions) were formulated. The nine possible combinations from three variables of the process parameters (drug ratio, pressure, temperature) were tested. The batch with 1:2 (Zidovudine–PLLA) ratio, at 45 °C and 85 bar gave 91.5% yield, with 40% drug content. Intestinal permeability studies revealed drug permeability of approximately 9.9%, which was higher than that of pure Zidovudine (3.8%).	[102]/ 2015
PCA	PLLA; 50	Insulin	Insulin-loaded PLLA porous MPs with sizes of ~4 µm were produced. The insulin encapsulation was found to be the highest with the lowest concentration of drug (5 wt. %). The physicochemical characterization revealed no major chemical changes in insulin but only minor changes in the secondary structure. The hypoglycemic activity was retained and sustained released was observed.	[103]/ 2015
SAS	PLLA; n/d.	17α-Methyltestosterone	Drug-loaded particles were produced and the in vitro drug release behavior was investigated. The study shows the use of SAS for the encapsulation of a hormone in biodegradable systems. The investigation leads to the conclusion that increasing the drug ratio concerning the polymer the drug entrapment increased but also led to aggregation in the final product.	[104]/ 2016
SAILA	PLLA; 28 PLGA; 20	Piroxicam	The drug encapsulation capacity of PLLA and PLGA was compared using piroxicam as the model drug. Particles of 1.53 μm ± 0.53 size were obtained from PLGA and broader distribution was obtained from PLA with 1.76 μm ± 1.05 size particles. Preliminary encapsulation studies revealed an EE of 60% and controlled release of up to 5 days.	[105]/ 2016
Rapid expansion of subcritical solution	PLLA; 4.7	Tetrahydro curcumin	Various drug:PLLA ratios on the particle morphology and stability of drug after processing were investigated. The average particle size was ∼80 to 110 nm and the loading capacity varied between ∼13 and 25%. It was shown that the loading capacity was directly proportional to the expansion temperature and the drug:polymer weight ratio, but inversely proportional to the expansion temperature. While the size of the particles had no major effect of process conditions.	[106]/ 2016
SAS	PLLA; n/d	Tamoxifen citrate	Encapsulation of tamoxifen citrate into PLLA particles was studied resulting in 280 nm size particles and in a high EE of 94%. Compared to the conventional encapsulation process, the SAS process provided the advantage of single-step processing.	[107]/ 2017
SAILA	PLGA (75:25); 20	Piroxicam; indomethacin; diclofenac	Various drug-loaded PLGA particles were produced with narrow distributions and sizes ranging from 0.3 to 2.5 µm. A higher drug ratio, temperature and pressure resulted in co-precipitation of the drug and polymer. A low EE was observed for the drugs that had more solubility in anti-solvent medium and also for higher process temperature.	[108]/ 2017
SAS	PLLA; 100	Gefitinib	The production of gefitinib (GFB) loaded PLLA particles resulted in being influenced by the solution flow rate affecting the loading of the GFB. The highest drug loading obtained with optimized conditions was 15.8% with an EE of 94% in spherical particles with D_50_ of 2.48 µm. GFB loaded MPs revealed a lower crystallinity compared to raw GFB. A sustained release over the time was measured which then was complemented with the in vitro anti-tumor experiments, where the encapsulated particles were more effective than raw GFB.	[109]/ 2017
PCA	PLLA; 50	Insulin	Preparation of insulin-loaded and -unloaded porous PLLA NPs at 80 bar and 35 °C (INS) with a solution flow rate of 4 mL min^−1^. The INS loading was determined to be 4.85% and the EE 97%. The particles revealed a porous, rough surface and sizes of 16 ± 3.14 µm for PLLA PM and 19.03 ± 3.05 µm for INS-PLLA PM as mean geometric diameter. The authors showed in vivo hypoglycemic effects for pulmonary delivery of INS-PLLA PMs compared to an untreated group supported with sustained release.	[110]/ 2019
SAS	PLLA; n/d	5-Fluorouracil	The production of pure drug particles and drug-loaded PLLA particles yielded particles with sizes from 220 to 670 nm depending on the process conditions. The highest drug loading capacity obtained was of 42% at 50 °C and 120 bar pressure. The organic solution mixture also influenced the particles sizes.	[111]/ 2019

Abbreviations: Aerosol solvent extraction system (ASES); compressed anti-solvent (PCA); gas anti-solvent (GAS); microparticle (MP); nanoparticle (NP); n/d—not determined; n/a—not applicable; poly(L-lactic acid) (PLLA); poly(D-lactic acid) (PDLA); poly(D,L-lactic acid) (PDLLA); poly(glycolic acid) (PGA); poly(lactic-*co*-glycolic)acid (PLGA); poly(ethylene glycol) (PEG); poly(caprolactone) (PCL); rapid expansion of supercritical solution (RESS); rapid expansion of supercritical solution in liquid solvent (RESOLV); supercritical assisted atomization (SAA); supercritical assisted injection in liquid antisolvent (SAILA); supercritical anti-solvent (SAS); supercritical anti-solvent precipitation with enhanced mass transfer (SAS-EM); supercritical fluid emulsion extraction-continuous (SFEE-C); solution-enhanced dispersion by supercritical fluids (SEDS); encapsulation efficiency (EE).

**Table 3:** Overview of loaded PLA/PLGA particles obtained using the supercritical fluid emulsion extraction (SFEE) method during the purification process.

Technique	Polymer; Molar Mass (kDa)	Cargo	Overview	Ref/Year
SFEE	PLGA (50:50); 42–65	Lysozyme	The influence of various emulsion technique parameters on the morphology of lysozyme loaded PLGA_(50/50)_ particles was studied. The particle sizes ranged from 0.1 to 1.7 µm. Among three different encapsulation methods, the in situ suspension emulsion was found to be most efficient with an EE of 48.5%.	[114]/ 2009
SFEE	PLGA (50/50); 42–65	Ketoprofen	Encapsulation of ketoprofen into PLGA particles applying first a conventional emulsion technique and subsequently SFEE was examined. Particles revealed a final particle size of 100–200 nm. The higher ketoprofen content reduced the stability and EE over time. Suspended co-formulation was speculated to be metastable and provided important information for the limit of the overloaded formulations.	[115]/ 2009
SFEE	PLGA (75/25); 60–120	Piroxicam; diclophenac sodium	Single and double emulsion techniques were compared with varying the emulsion process parameters in the encapsulation of piroxicam and diclophenac sodium into PLGA particles. Drug-loaded particles with sizes between 1 to 3 µm were obtained with EEs of 88% and 97% for diclofenac sodium and piroxicam, respectively. The SFEE proved to be an efficient technique for the production of drug-loaded particles.	[116]/ 2010
SFEE	PLGA (75/25); 20	Retinyl acetate	The production of retinyl encapsulated PLGA (75:25) NPs via batch and continuous mode of operation was tested. The continuous mode was more efficient and yielded 3.3 to 4.5 µm sized particles with high EEs of 80 to 90%. In this study, the mass transfer phenomenon of the process was exploited.	[117]/ 2011
SFEE-C	PLLA; 28 PLGA (75/25); 20	-	PLLA, PLGA (75/25) and PCL particles were prepared by the application of various process parameters. Operating conditions of 38 °C, 80 bar and L/G ratio of 0.1 resulted in particles of PLA, PCL and PLGA with a mean size of 233 nm, 342 nm and 212 nm, respectively. The influence of various polymer concentrations on the final particle sizes and distributions were studied.	[118] 2013
SFEE-C	PLGA (50/50); 44	Insulin	Insulin-loaded PLGA MPs were produced using double emulsion and tested on rat embryonic cell for viability and drug release studies. Particles sizes resulted between 2 to 3 µm and revealed no degradation of the insulin which maintained further the activity in the cells.	[64]/ 2013
SFEE	PLGA (75/25); n/d	Celecoxib	Celecoxib loaded PLGA (75/25) MPs of 10.5 µm size were produced with a drug loading of 10.9%. These particles were found to be more stable and had higher accumulation levels of celecoxib to the targeted area compared to the conventional particles.	[119]/ 2013
SAA and SFEE-C	PLGA (50/50); 38–54	Hydrocortisone acetate	The rapid recrystallization of hydrocortisone acetate while encapsulation into PLGA and continuous emulsion processing were resolved and particles of ~3 µm with an EE of 75% were obtained.	[120]/ 2013
SFEE	PLLA; 60 PLGA (75/25); 20	β-Carotene, α-tocoferol, rosmarinic acid	Drugs were encapsulated in the polymeric carriers to improve their shelf life and stability. The solvent was removed at 80 bar and 37 °C. The encapsulation of β-carotene in PLGA, as well as co-encapsulation of α-tocoferol and β-carotene, was possible resulting in range of sizes 0.3 to 4.3 µm (for PLLA and PLGA) with improved shelf life of the drugs. Rosmarinic acid encapsulation was limited to low efficiencies.	[113]/ 2019
SFEE	PLLA; n/d	Rhodamine B	Rhodamine B loaded emulsion microbeads were prepared and exposed to SFEE. The size of the microbeads was 1 ± 0.2 µm. Human monocytes showed uptake of loaded microbeads. The technique provided an excellent alternative to the conventional technique with improved product quality and reduced residual solvent.	[11]/ 2019

Abbreviations: Microparticle (MP); nanoparticle (NP); n/d—not determined; n/a—not applicable; poly(L-lactic acid) (PLLA); poly(D-lactic acid) (PDLA); poly(D,L-lactic acid) (PDLLA); poly(lactic-*co*-glycolic)acid (PLGA); poly(caprolactone) (PCL); supercritical fluid emulsion extraction-continuous (SFEE-C); solution-enhanced dispersion by supercritical fluids (SEDS); supercritical assisted atomization (SAA).

## Abbreviations

CO_2_	Carbon dioxide
CH_2_Cl_2_	Dichloromethane
DMSO	Dimethyl sulfoxide
DSC	Differential scanning calorimetry
EE	Encapsulation efficiency
FA	Folate
FDA	Food and drugs association
FTIR	Fourier transform infrared spectroscopy
5-FU	5-Florouracil
GA	Glycolic acid
GAS	Gas antisolvent solution
G/L	Gas/liquid
GMS	Glyceryl monostearate
L/D	Length/diameter
LA	Lactic acid
LC	Loading capacity
MP	Microparticle
μm	Micrometer
Mn	Molar mass
NP	Nanoparticle
Nm	Nanometer
kDa	Kilodalton
P	Pressure
PCL	Poly(caprolactone)
PCA	Pressure compressed antisolvent
PEG	Poly (ethylene glycol)
PDLA	Poly (D-lactic acid)
PLLA	Poly (L-lactic acid)
PLGA	Poly (lactic-*co*-glycolic acid)
P_C_	Critical pressure
RESS	Rapid expansion of supercritical solution
RESOLV	Rapid expansion of supercritical solution in liquid
SAA	Supercritical assisted atomization
SAS	Supercritical antisolvent
SAILA	Supercritical antisolvent in liquid
SAS-EM	Supercritical antisolvent- enhanced mass transfer
SC-CO_2_	Supercritical carbon dioxide
SCF	Supercritical fluid
SDS	Sodium dodecyl sulfate
SEDS	Solution-enhanced dispersion solvent
SEM	Scanning electron microscopy
SFEE	Supercritical fluid emulsion extraction
T	Temperature
T_g_	Glass transition temperature
T_m_	Melting temperature
TAM	Tamoxifen
TEM	Transmission electron microscopy
